# Novel lytic bacteriophage AhFM11 as an effective therapy against hypervirulent *Aeromonas hydrophila*

**DOI:** 10.1038/s41598-024-67768-2

**Published:** 2024-07-23

**Authors:** Nithin Muliya Sankappa, Girisha Shivani Kallappa, Kushala Kallihosuru Boregowda, Namrutha Mandrira Ramakrishna, Prithvisagar Kattapuni Suresh, Dheeraj Shriraje Balakrishna, Krishna Kumar Ballamoole, Suresh Thangavel, Lopamudra Sahoo, Miles D. Lange, Michael B. Deshotel, Jason W. Abernathy

**Affiliations:** 1 Department of Aquatic Animal Health Management, College of Fisheries, Karnataka Veterinary, Animal and Fisheries Sciences University, Matsyanagar, Mangaluru, Karnataka 575002 India; 2 ARS Research Participation Program, Oak Ridge Institute for Science and Education (ORISE), Oak Ridge, TN 37830 USA; 3 Aquatic Animal Health Research Unit, United States Department of Agriculture, Agricultural Research Service, Auburn, AL 36832 USA; 4 Division of Infectious Diseases, Nitte University Centre for Science Education and Research, Mangaluru, India; 5 Department of Fish Genetics and Reproduction, College of Fisheries, Central Agricultural University (Imphal), Lembucherra, Tripura West, Tripura 799210 India; 6 United States Department of Agriculture, Agricultural Research Service, Harry K. Dupree Stuttgart National Aquaculture Research Center, Stuttgart, AR 72160 USA

**Keywords:** Aquaculture, Lytic phage, Hypervirulent *Aeromonas hydrophila*, Antibiotic resistance genes and biocontrol, Bacteriophage therapy, Bacteriophages, Bacterial infection, Ichthyology

## Abstract

Several farmed fish species, including carps, tilapia, salmon, and catfish, have experienced significant economic losses in aquaculture due to motile *Aeromonas* septicemia caused by *Aeromonas hydrophila*. In the present study, a novel lytic bacteriophage infecting hypervirulent *Aeromonas hydrophila* (vAh) was isolated and characterized. This is the first report of a phage against vAh. Phage AhFM11 demonstrated lytic activity against both vAh strains and the *A. hydrophila* reference strain ATCC 35654. The AhFM11 genome was sequenced and assembled, comprising 168,243 bp with an average G + C content of 41.5%. The genome did not harbor any antibiotic resistance genes. Genomic information along with transmission electron microscopy revealed that phage AhFM11 belongs to the *Straboviridae* family. Therapeutic application of monophage AhFM11 in fish showed 100% survival in injection, 95% in immersion and 93% in oral feeding of phage top-coated feed. Fish and chicken meat spiked with *A. hydrophila* and phage showed significant reduction of *A. hydrophila*. These findings support that phage AhFM11 can be used as a biocontrol agent against vAh as an alternative to antibiotics.

## Introduction

Aquaculture is one food sector that continues to be a significant contributor to the global food supply with production surpassing 122.6 million tons in 2022^1,2^. The largest contributions are from finfish which account for over 60% of the market and include carp followed by tilapia and catfish^1–4^. This sector plays a crucial role in food security, especially in developing nations, where it contributes nearly 25% of the total food supply and is rapidly increasing^2^. To answer this increasing demand for aquaculture products, the prevailing trend of aquaculture practitioners has been to grow fish at higher densities leading to higher production and profits. Unfortunately, these density increases are correlated to higher levels of disease transmission due to increased contact between the fish and higher levels of pathogens that reap nutrients from uneaten pelleted feed^4–10^. The yearly economic losses caused by these diseases range from $6–$10 billion USD annually^11–13^.

Diseases with Gram-negative bacteria as the causative agents occur at the highest frequencies in aquaculture^14^ and include motile *Aeromonas* septicemia (MAS) caused by *Aeromonas hydrophila* and hypervirulent *A. hydrophila* (vAh). MAS has been the most prevalent disease reported in freshwater aquaculture in recent years^4,14–19^. *A. hydrophila* is ubiquitous and clinical symptoms include edema, hemorrhage, scale and fin erosion, tail rot and ulcerative syndrome^6,20–22^. Amplifying the problems associated with *A. hydrophila* infections is the emergence of antibiotic-resistant strains which pose a significant threat to the sector's sustainability^7,9,12,23–27^. Furthermore, the recent emergence of vAh strains in carp and catfish from China and Vietnam has alarmed the aquaculture community due to their severe impact on fish health and production and has caused several million dollars of economic loss since 2009^15,16,28^. To ameliorate these bacterial infections, feed that has been top coated with antibiotics is administered^29^. However, within the United States, the only readily available FDA-approved finfish antibiotic for use against vAh is florfenicol^30^. Six genes are already known to confer resistance to florfenicol and *Aeromonas* species’ have been documented to carry at least three thus emphasizing the need for antibiotic alternatives^31–33^.

Growing concerns about antibiotic resistance have spurred the search for eco-friendly and cost-effective alternatives to prevent and treat diseases in aquaculture. While vaccines offer a preventive approach, their application in juvenile fish and farmed crustaceans/mollusks is often impractical. Fortunately, numerous biological alternatives to antibiotics exist, including lytic bacteriophages (phages), endolysins, biotics, and quorum sensing mechanisms^5,8,34–36^. Among these, lytic phages are the naturally occurring predators that target and kill bacteria rapidly^37^. Although they are currently underutilized, their therapeutic and prophylactic potential combined with their affordability makes them a valuable addition to mitigation efforts against the prevention and spread of antibiotic resistance^34,35,38,39^. Investigational research on lytic phages that target *A. hydrophila* have been studied through the injection route in zebrafish, striped catfish, brocade carp, crucian carp, loach, eels and tilapia^40–44^; by immersion in tilapia, zebrafish, white leg shrimp, and cockles^42,45–47^; and by oral feeding in eels and tilapia. In each study, the phage treated animals exhibited better survival when challenged with bacteria^40,44^. Here, we describe the methods used to discover a new *A. hydrophila* phage, AhFM11, and investigated its ability to protect rohu (*Labeo rohita*) from vAh. Additionally, we explored cryopreservation methods, sequenced and annotated the genome, as well as demonstrated its ability to decontaminate *A. hydrophila* from meat products.

## Results

### Environmental isolation, host range, one step growth curve and adsorption of phage

Isolation of vAh phages was carried out by collecting river water samples from different locations in Karnataka, India. Two phages named AhFM10 and AhFM11 were isolated using vAh strains HypAh-12 and HypAh-20, respectively as host bacteria. The location, titer value, and water sample source are referenced in Table 1 and Supplemental Fig. 1. To classify the phages as broad or narrow spectrum, *Aeromonas* spp. and non-*Aeromonas* spp. were used to study their host ranges. Defining the host ranges allows for the proper selection of phages for use in different therapies. Phages AhFM11 and AhFM10 had lytic activity against 84 and 53 *Aeromonas* isolates, respectively (Supplemental Table 1). AhFM11 had the largest host range and was further used for phage stability tests, one step growth curves, adsorption tests, genomic characterization, and monophage therapy in fish and meat products.

**Table 1:** Phage name, location, titer values, host, and source of water for isolation of *Aeromonas hydrophila* phages.

Phage name	Isolation Date	Latitude longitude	Titer (PFU/ml)	Water sample source	*A. hydrophila* Host Strain
AhFM10	25-02-2019	14.646 74.321	1.58 × 10^10^	Creek	HypAh-12
AhFM11	25-02-2019	14.448 74.446	1.61 × 10^9^	River	HypAh-20

**Figure 1:**
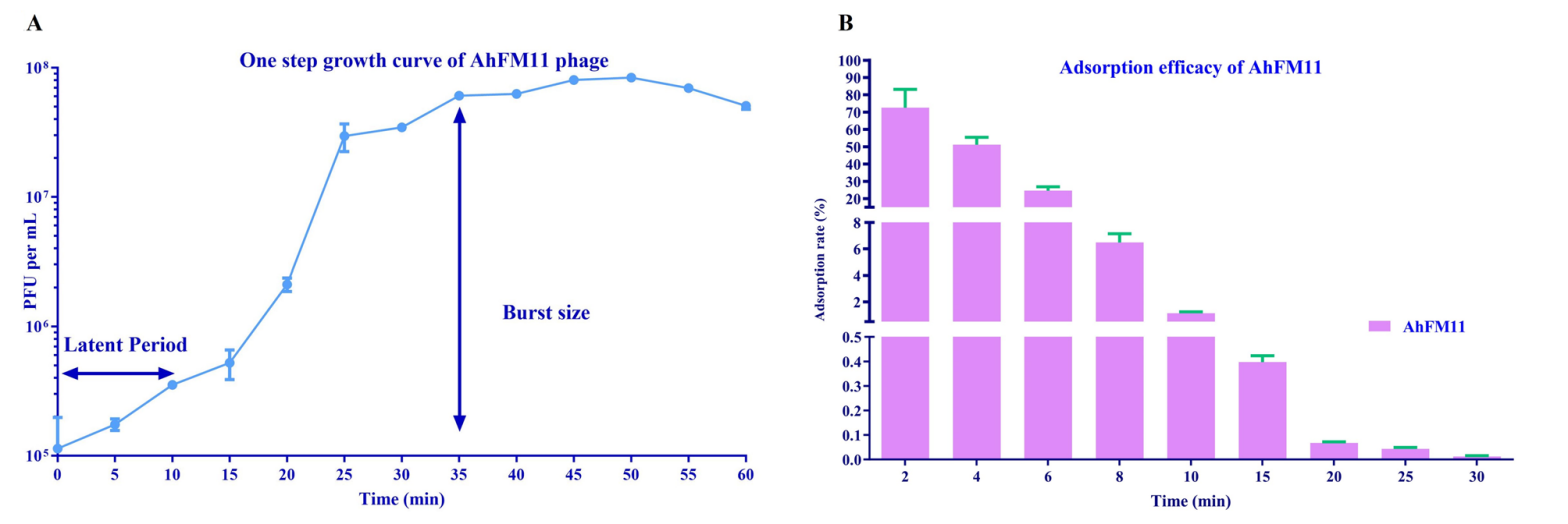
In vitro growth characteristics of phage AhFM11 (**A**) one-step growth curve showing the latent period and burst size of the phages (**B**) adsorption efficacy. This experiment was carried out at MOI 0.001. Error bars represent the Mean ± SD (n = 3).

One-step growth curves were performed in TSB at 28 °C to determine the latent period and burst size of phage AhFM11 (Fig. 1A). AhFM11 had a latent period of 10 min and burst size of 378 ± 49 PFU/cell. The adsorption efficiency exhibited by AhFM11 was 99.7% adsorbed within 30 min and complete adsorption (no free phages) by 40 min (Fig. 1B).

### AhFM11 plaque morphology, transmission electron microscopy (TEM), and phage stability assays

**Figure 2:**
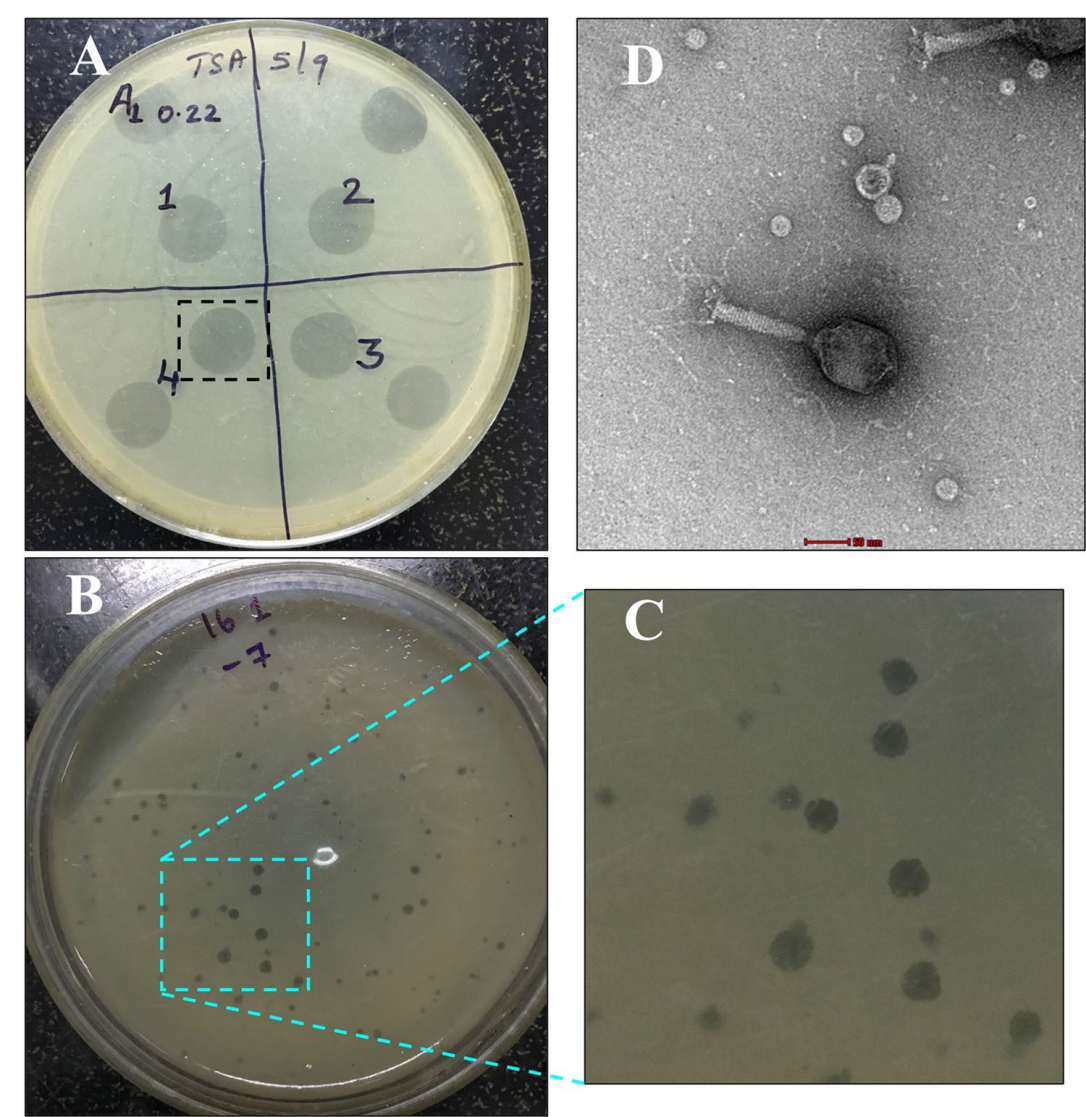
Phage (AhFM11) characteristics on agar plate, soft overlay method and transmission electron microscopy. (**A**) Spot tests for phage AhFM11 on hypervirulent *Aeromonas hydrophila* HypAh-20 and the spot 4 (black dotted rectangle) was used for further passage to get the titer value for AhFM11. (**B**,**C**) Plaque morphology of AhFM11 on TSA soft agar plates showing 1–3 mm plaque diameter. (**D**) Transmission electron microscopy image negatively stained with 2% uranyl acetate showing single phage AhFM11 (Bar = 50 nm) with its icosahedral head along with sheathed tail tube and its branched tail fibers.

AhFM11 formed a clear zone in the soft agar overlays with a plaque diameter ranging from 1–3 mm (Fig. 2A, B, C). TEM was employed to determine morphology and revealed that AhFM11 has an icosahedral head length of 96.6 ± 8.8 nm and width of 68.7 ± 3.4 nm, a tail length of 100.2 ± 9.3 nm, a collar length of 16.5 ± 3.2 nm and a total length of 211.4 ± 11.5 nm long (Fig. 2 D). Based on the morphological analysis in combination with NCBI BLAST results of the nucleotide sequence, AhFM11 was assigned to the family of *Straboviridae*.

During the 30% glycerol storage stability studies, the samples of AhFM11 stored at − 80 °C and − 20 °C significantly retained their titer values even after 60 days of storage whereas phages stored without glycerol lost their activity (Supplemental Fig. 2A). Samples stored at 0 °C and 4 °C showed a slight but significant decrease in their titer values and AhFM11 lost ~ 50% its titer value at 28 °C and ~ 75% of its titer value at 37 °C (ANOVA, *p* < 0.05) when compared with the phages stored with glycerol at − 80 °C and − 20 °C. The phage titer decreased over a period of 60 days when stored at 4 °C in different salinity concentrations (0.1, 0.5, 1.0, 2.0 and 3.5%). In comparison to phages stored in 1% salt, which was used as a baseline control, there was a significant difference in the 0.1 and 3.5% salinity groups whereas there were no significant differences in the rest of the salinities (Supplemental Fig. 2B). Hence, the phage was stable at wide range of salinities suggesting it will remain functional in both freshwater and estuarine waters. The phage lost its activity at acidic pH (2 and 4) and alkaline pH (10 and 12) but remained functional from pH 5–8 (Supplemental Fig. 2C). AhFM11 retained the highest titer values when stored in PBS and SM buffers followed by chloroform. However, AhFM11 significantly lost lytic functionality when exposed to phenol, propanol, ethanol, and acetone (Supplemental Fig. 2D).

### Whole genome sequencing of AhFM11 and phylogenetic analysis

**Figure 3:**
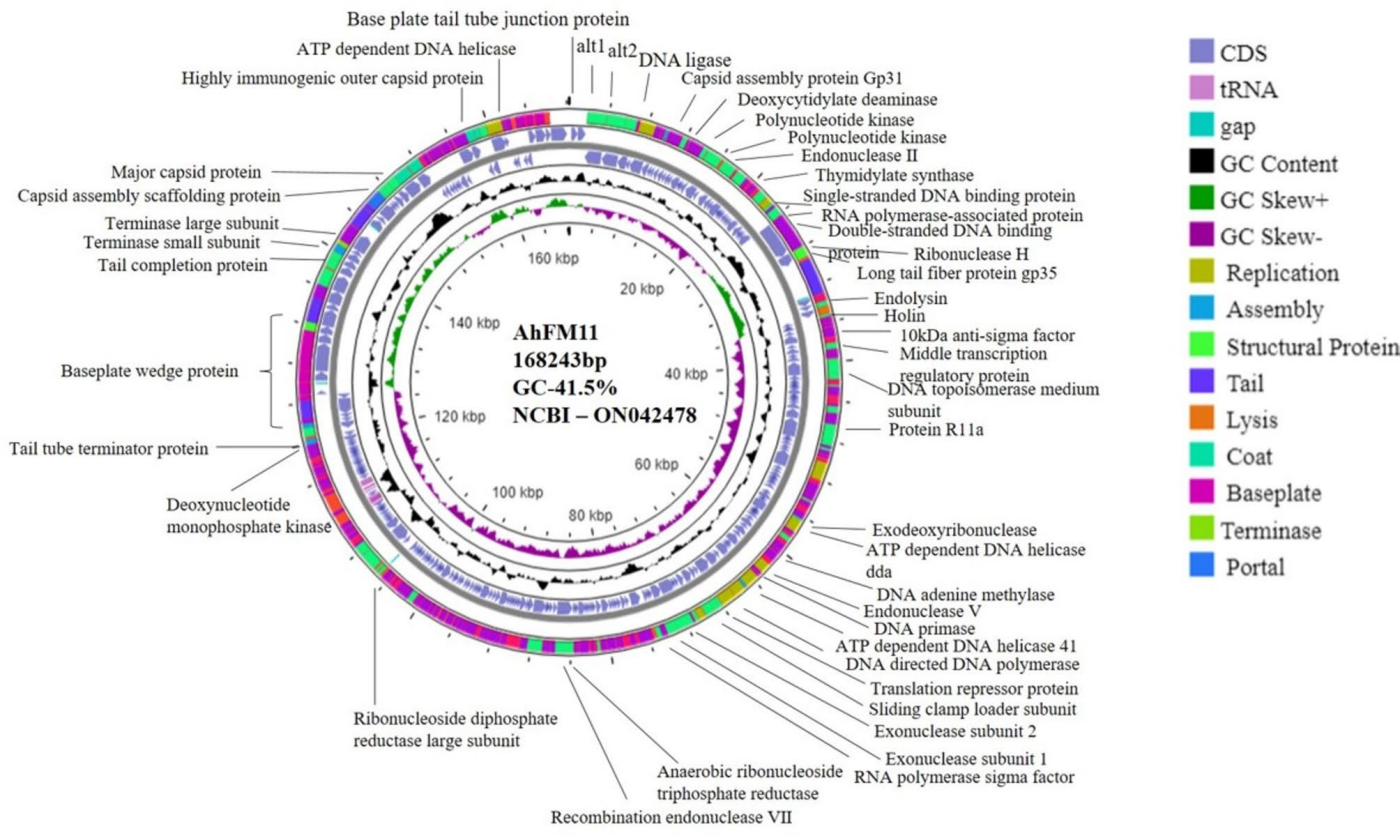
Circular genome map of phage AhFM11 against the hypervirulent *Aeromonas hydrophila*. The ORFs are marked with arrows indicating the direction of transcription. The ORFs encoding putative proteins for which functions could be predicted are color-coded and labelled on the figure.

**Figure 4:**
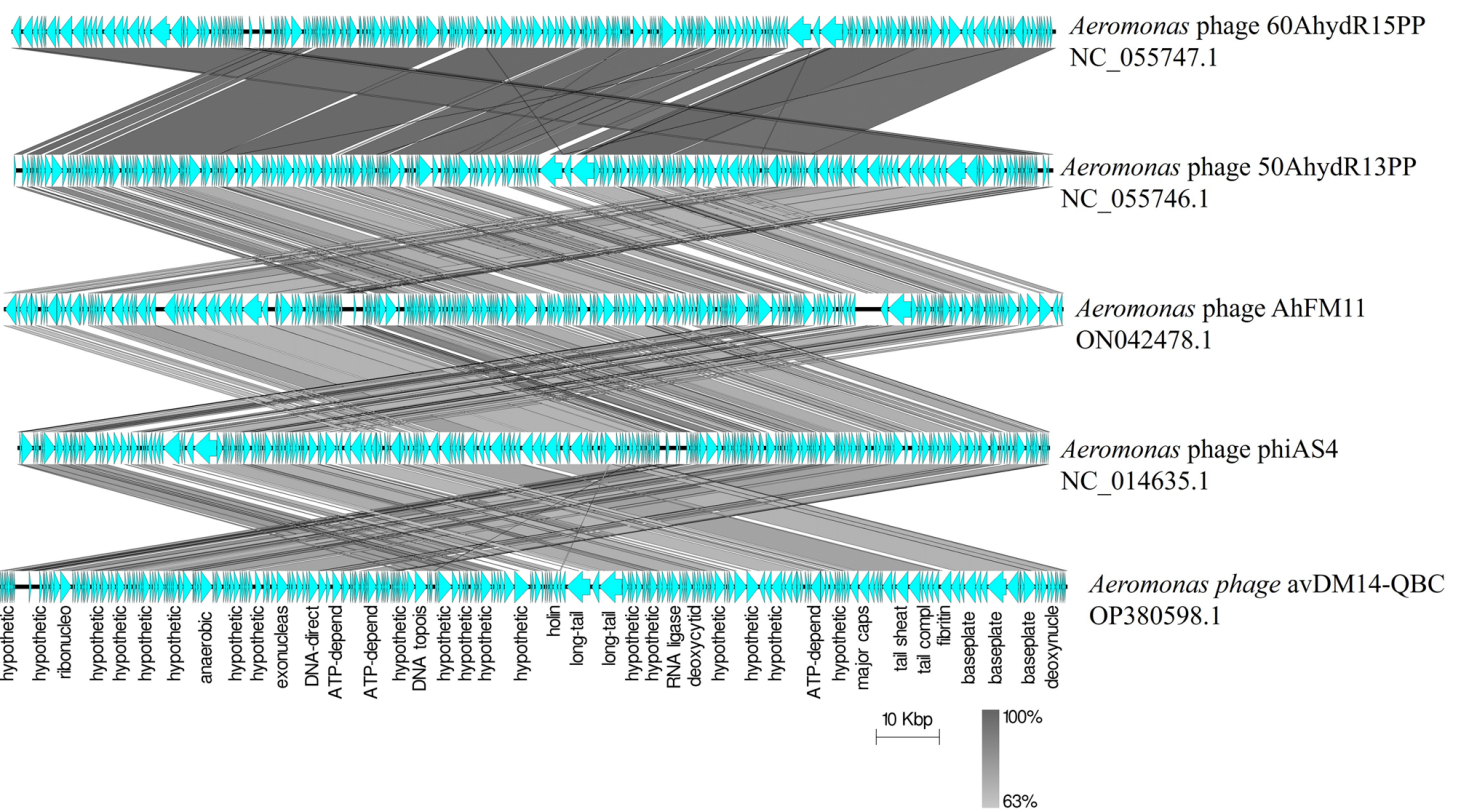
Comparative analysis of four phages was conducted using Easyfig. Coding domain sequence (CDS) are shown as arrows to indicate the direction of transcription and are mentioned in the bottom legend in accordance with their predicted functions. The percentage of sequence similarity is shown as the intensity of the gray to black color.

**Figure 5:**
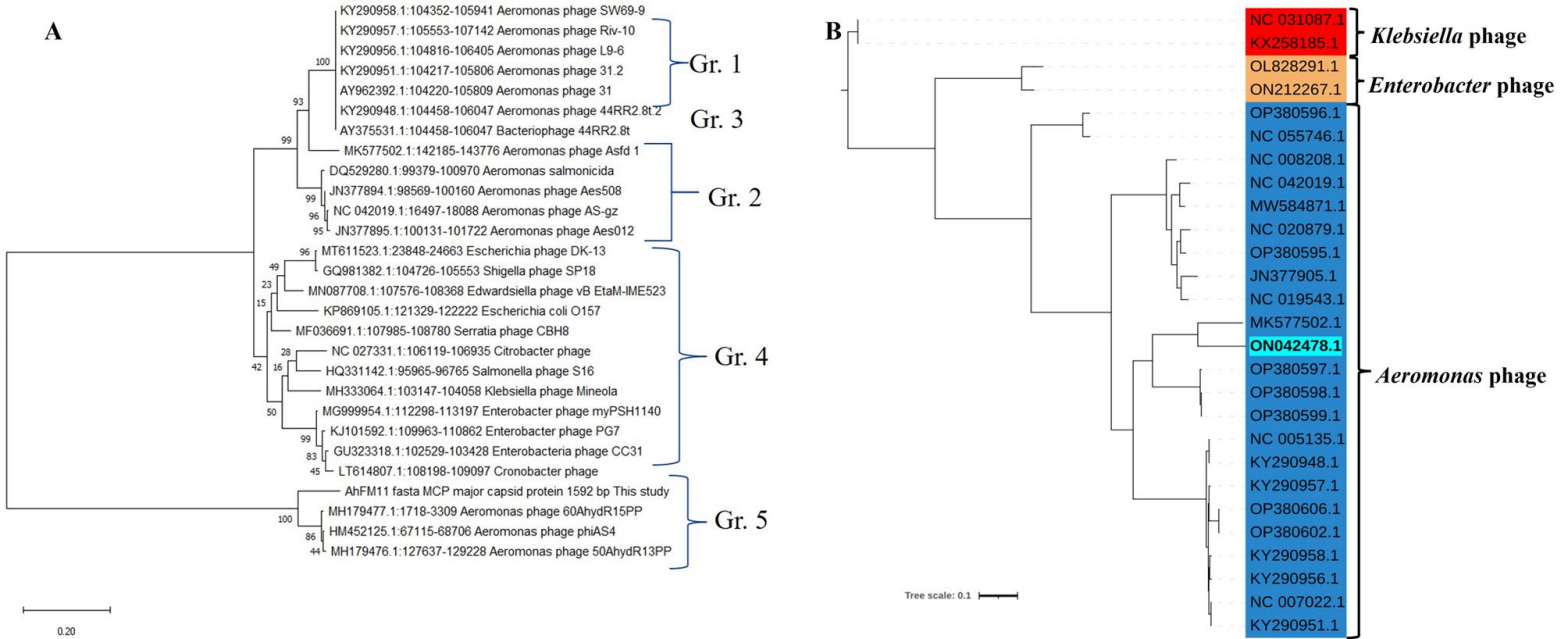
Phylogenetic tree of (**A**) *MCP* gene of phages constructed using the Maximum-likelihood method with a bootstrap value of 1000 replicates. A total of 28 nucleotide sequences were included for the analysis including AhFM11 (1-Present study), *A. hydrophila* phage (11), *A. salmonicida* (4) and Enterobacteriaceae phages (12). (**B**) Phylogenetic tree of whole-genome sequences of phages were aligned using MAFFT v7.3, the tree was constructed using online software by interactive tree of life (iTOL) and the bolded ON42478.1 indicates the phage AhFM11 isolated from the present study.

Whole genome sequencing, assembly, annotation, and phylogenetic tree construction was completed on AhFM11. AhFM11 has a medium-sized phage genome containing 168,243 bp with a G + C content of 41.5% (Fig. 3). We identified 265 coding sequences (CDS), and functional predictions were made for 72 ORFs and 16 tRNAs. The remaining CDS were classified as "hypothetical proteins" with unknown functions. The length of nucleic acid CDS varied from 75 to 3672 bp and encoded notable putative proteins: RNA polymerase, NAD (+)-arginine ADP-ribosyl transferase, ribonucleoside-diphosphate reductase, helicase, kinase, DNA polymerase, topoisomerase, protease, and DNA ligase. Comparative genome analysis was performed with four phages possessing > 85% similarity namely avDM14-QBC (Accession #OP380598.1), 50AhydR13PP (Accession #MK179477.1), 60AhydR15PP (Accession #MH179476.1) and *Aeromonas* phage phiAS4 (Accession #HM452125.1). All these phages contain similar genome organization as to our phage AhFM11 (Fig. 4 and Supplemental Table 2). At the nucleotide level, the phage genome shared the highest homology with *Aeromonas* phage Asfd_1 (accession number: MK577502.1) with an identity of 84.47%. Maximum-likelihood trees of major capsid proteins also indicated that the phage is closely related to the 50AhydR13PP (MK179477.1), 60AhydR15PP (MH179476.1) and *Aeromonas* phage phiAS4 (HM452125.1), unclassified Secunda5virus of the family *Straboviridae* (Fig. 5A). As shown in Fig. 5B, whole genome analysis revealed that the phage AhFM11 was closely related to the *Aeromonas* phage Asfd_1 (Accession #MK577502.1), forming its own grouping withing the *Aeromonas* phages. Separate clades were also formed between the *Aeromonas* phages, *Enterococcus* phages, and *Klebsiella* phages. It did not encode any known bacterial virulence-related proteins or antimicrobial resistance genes, based on the predictions by the VFDB and CARD database. The PHACTS tool predicted the phage lifestyle to be lytic. The full sequence and annotation of phage AhFM11 were submitted to the NCBI and assigned accession number ON042478.1.

**Table 2:**
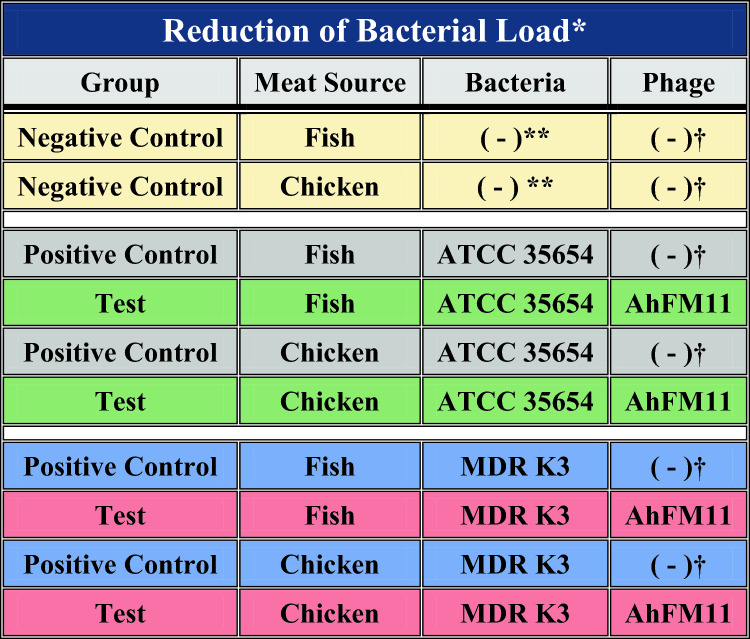
Food decontamination efficacy of phage AhFM11 with different treatment groups in fish and chicken meat to test the reduction of bacterial load.

*Colors are coordinated to match Supplemental Fig. 3.

**TSB broth added in place of Bacteria.

^†^SM buffer used in place of bacteriophages.

### In vivo experiments with phage AhFM11 against *Aeromonas* infection

**Figure 6:**
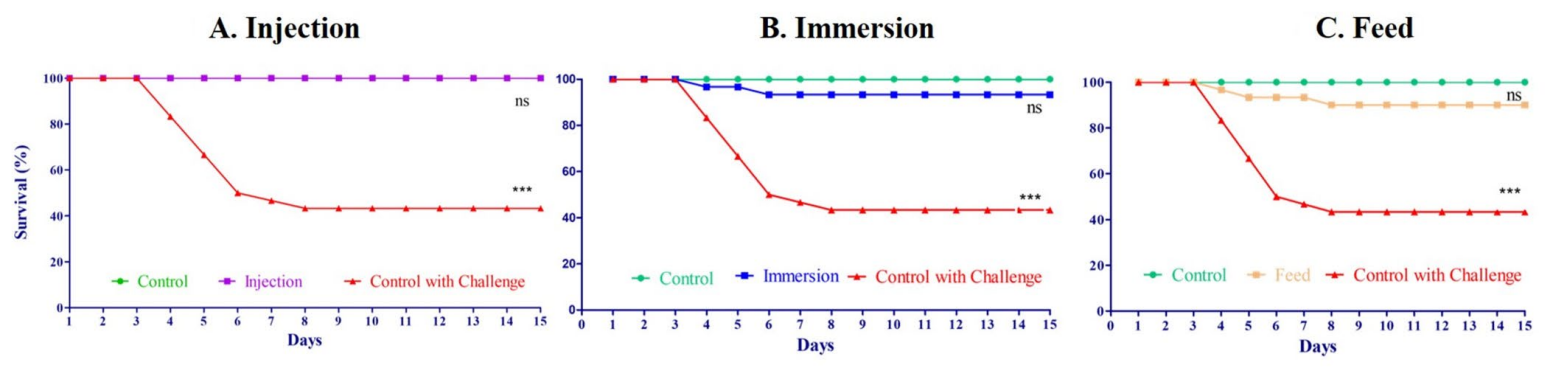
Therapeutic protective effects of phage AhFM11 in vivo. (**A**) injection, (**B**) immersion and (**C**) phage impregnated feed groups. Mortality was recorded daily for 15 days, and cumulative percent survival rate was calculated.

For the in vivo efficacy tests, all "Challenged” groups received the predetermined LD_50_ (2.42 × 10^6^ CFU/fish) intraperitoneal injection of vAh (HypAh-20). In the “Injection Group,” rohu carp received an injection (100 µL) containing 1.55 × 10^6^ PFU/fish of AhFM11 2 h post infection and demonstrated 100% survival after the 15-day challenge. In the “Immersion Group,” fish were groups receiving AhFM11 had the following survival percentages: 100% when delivered by intraperitoneal injection (Fig. 6A), 95% with the immersion (Fig. 6B), and 93% with top-coated feed (Fig. 6C). These results indicated that AhFM11 can be injected or ingested by fish yet retain its lytic capacity. Based upon these results, phage AhFM11 can be delivered efficiently through injection, immersion, and oral feeding to treat *A. hydrophila* infections.

### Food decontamination efficacy of phage AhFM11 on *Aeromonas* hydrophila

Fish and chicken meat samples were artificially contaminated with MDR K3 or ATCC 35654 *A. hydrophila* and treated with phage AhFM11 (MOI = 1000) as described in the Methods section. Significant reductions (*p* < 0.05) in bacterial counts were observed at all time points (24, 48, 72, and 96 h) compared to untreated controls (details provided in the Methods section). For fish meat samples contaminated with ATCC 35654, the application of phage AhFM11 resulted in reductions of the bacteria.

For the fish meat samples, the colony counts of ATCC 35654 *A. hydrophila* treated with phage AhFM11 resulted in 2, 1.5, 1.7 and 2.3 log_10_ CFU/g reductions at 24, 48, 72, 96 h when compared with the untreated control group (Supplemental Fig. 3A). The colony counts of ATCC 35654 *A. hydrophila* in chicken meat samples treated with AhFM11 revealed drastic reductions of 2.9, 4.2, and 3.2 log_10_ CFU/g at 24, 48, and 72 h; however, the 96 h timepoint was reduced below the detection limit. (Supplemental Fig. 3B).

When MDR K3 *A. hydrophila* was used to contaminate fish meat, AhFM11 treated samples had a 1.2 and 0.8 log_10_ CFU/g reduction at the 48 and 96 h timepoints. At the 24 h timepoint, there was < 1 log_10_ CFU/g reduction and the 72 h had a 1.5 log_10_ CFU/g reduction (Supplemental Fig. 3C). The chicken meat contaminated with MDR K3 and treated with AhFM11 had 0.3 log_10_ reduction at 24 h; 1.2 log_10_ CFU/g reduction at 48 h; 0.2 log_10_ CFU/g reduction at 72 h, and no reduction at 96 h. The positive growth control sample at 72 and 96 h was same as the phage treated (Supplemental Fig. 3D).

In the case of fish meat, ATCC 35654 *A. hydrophila* treated with phage AhFM11 showed a 0.72 log_10_ PFU/g increase in phage titer value at 24 h when compared to only phage treated fish meat sample. Later timepoints showed no significant increase in phage titers (Supplemental Fig. 4A). Chicken meat samples treated with ATCC 35654 *A. hydrophila* and phage AhFM11 showed a significant increase in phage titer with 0.44, 0.40, and 0.46 log_10_ PFU/g at 24, 48 and 96 h respectively, except 72 h (Supplemental Fig. 4B). Whereas in fish meat sample treated with MDR K3 *A. hydrophila* with phage AhFM11 showed a significant increase of 0.107, 0.139, 0.363 and 0.914 log_10_ PFU/g increase in phage titer value at 24, 48, 72 and 96 h time points, respectively (Supplemental Fig. 4C). Similarly in chicken meat samples showed an increase in phage titer value with 0.885, 0.396, 0.408 and 1.00 log_10_ PFU/g at 24, 48, 72 and 96 h timepoints, respectively (Supplemental Fig.4D).

## Discussion

Increasing fish density practices of modern aquaculture, coupled with unregulated antibiotic administration in some countries, and climate change has led to a surge in disease outbreaks and increasing prevalence of antimicrobial resistance (AMR). *A. hydrophila* as a causative agent is documented as the most prevalent finfish disease and there are no commercially available vaccines against *A. hydrophila* in India or the United States^38^. Even if a protective *A. hydrophila* vaccine were available, their use in aquaculture is often unrealistic due to the labor costs associated with administering a vaccine by hand or the exorbitant price of automated mechanical injectors. An additional concern related to *A. hydrophila* is the identification of AMR and bacterial biofilm formation in recirculating aquaculture systems that compromise the effectiveness of antibiotics ^18,48–50^. These factors underscore the critical need for alternative approaches to manage *A. hydrophila* infections in fish that are simple, cost-effective, and environmentally friendly. Phage therapy presents a promising solution in this regard.

In the present study, phages AhFM10 and AhFM11 were isolated from river waters and showed significant lytic activity against the vAh strains HypAh-12 and HypAh-20. HypAh-12 isolated from rohu carp and HypAh-20 isolated from koi produce similar clinical symptoms (reddening, enlargement of abdomen and petechial haemorrhages) of vAh infected carps and catfishes in China and the USA^7,8,21^. The result of the plaque spot tests showed that phage AhFM10 and AhFM11 can lyse 53 and 84 isolates respectively, indicating a broader lytic efficacy in case of AhFM11 (Supplemental Table 1). The host range determination revealed that efficiency to lyse *A. hydrophila* was characterized into broader lytic efficacy and has the potential to be used for phage therapy as reported by several studies^5,39,51^. However, phage AhFM11 was selected due to its broader host range and to explore its potential as a therapeutant in aquaculture and food decontaminant. TEM images of AhFM11 indicated a morphology with an icosahedral head attached to a long tail that is characteristic of the *Straboviridae* family. Most of the lytic bacteriophages reported against *Aeromonas* spp. have been described as tailed phages belonging to the family *Myoviridae*^5,6,8,32,39,51–57^, and most recently, this has been re-classified as belonging to family *Straboviridae*^58^.

To be suitable for use in therapy and on food products, it is essential to characterize the physiochemical limitations of AhFM11^47^. Under the test conditions used in this study, AhFM11 is relatively stable with 30% glycerol at − 80 °C and − 20 °C for at least 60 days. Storage at 0 °C, 4 °C, and 28 °C without glycerol exhibited decreasing lytic activity with increasing temperature (Supplemental Fig. 2A). Phages completely lost their lytic capacity after 60 days of incubation without glycerol at − 80 °C, − 20 °C, and 37 °C. When evaluating the effect of salinity on AhFM11 function, phages stored at 4 °C in 0.5%, 1.0%, and 2.0% salinity retained similar lytic capacity in comparison to phages stored at low temperatures in glycerol. However, salinities of 0.1% and 3.5% showed a dramatic reduction in lytic function. There are reports of *A. salmonicida* phages which were stable up to 37 °C and in 3.5% salinity for a duration of 49 days^47,56^. AhFM11 was stable for 45 days at 37 °C and in 3.5% salinity suggesting this phage can be used for both freshwater and estuarine waters. Several factors are responsible for the natural progressions of phage infection including attachment, penetration, and multiplication; among the different environmental factors, temperature and pH are the main physiochemical parameters that play vital roles in phage therapy within an aquaculture environment^41^. In the present study, AhFM11 was the most functional, in terms of lytic plaque formation, at pH 6, 7, and 8. This demonstrated that AhFM11 has excellent stability around neutral pH, which is the optimum range for warm water fish farming. Generally, pH values < 5 and > 8 impair the lytic activity of phages which was also evident in the present study^41,47,56^. To determine whether AhFM11 contains structural lipids that are essential to its life cycle, chemical stability tests were performed with chloroform, phenol, ethanol, propanol, SM buffer, PBS and Diethyl ether. There was a 1 log reduction in the titer value after 10 min and 2-log reduction 24 h post chloroform treatment at room temperature. The results demonstrate that AhFM11 is stable in chloroform and suggests the absence of structural lipids. Several studies correlate with these results and have reported that other *A. hydrophila* phages are stable in PBS, SM buffer, and chloroform, and less stable with diethyl ether^39,42,47,52,53,59,60^.

Genomic sequencing revealed that AhFM11 has a genome size of 168,243 bp and G + C content of 41.5%. Similar results were seen for phages AhSzq-1 (112,558 bp and 43.86%), AhSzw-1 (115,739 bp and 43.82%)^61^; 50AhydR13PP (144,979 bp and 41.10%), 60AhydR15PP (165,795 bp and 41.2%)^54^, and Akh-2 (114,901 bp and 45.22%)^41^. Nucleotide sequence analysis revealed and absence of known virulence and antibiotic resistance genes which further increases its potential as phage therapeutic and food decontaminant. Similar results were also confirmed by Kazimierczak et al.^54^ when they analysed the genome of 7 *A. hydrophila* phages. From these seven phages, three (25AhydR2PP, 50AhydR13PP, 60AhydR15PP) were described to have potential as a phage therapeutant due to a confirmed lytic life cycle and the absence of virulence and antibiotic resistance genes.

The novel phage, AhFM11, possesses the basic characteristics for a candidate therapeutic agent. Phages delivered through injection, immersion, and oral feeding routes in this study all exhibited remarkable protection against vAh infections. In this experiment, phage AhFM11 provided protection through injection (100%), immersion (95%) and oral feeding of top-coated feed (93%) and similar results were obtained by Cao et al.^52^. Numerous phage therapy studies have reported on the protective effects of *Aeromonas* phages against *A. hydrophila* but none have achieved the remarkable protection rate presented here^8,39,41,42,44,46–49,51,53–56,59–65^. Jun et al.^60^ reported that intraperitoneal injections with phages pAh1-C (3.0 × 10^7^ PFU/fish) and pAh6-C (1.7 × 10^7^ PFU/fish) had cumulative mortalities of 43.33% ± 2.89% and 16.67% ± 3.82%, respectively. When the fish were fed phage top-coated feed, the cumulative mortality rates were 46.67% ± 3.82% (pAh1-C) and 26.67% ± 2.89% (pAh6-C), which indicated that injection provided better protection than phage feeding. In the present study, we likewise observed better protection via the injection route in comparison to the oral feeding route.

During the food decontamination studies, fish meat samples treated with phage AhFM11 had 1.5 and 2 log reductions in CFU/g of meat when using the AMR strain MDR K3 and 2 and 2.3 log reductions when using ATCC35654. These results are consistent with a study carried out on lettuce leaves by Islam et al.^66^ where the antibacterial effectiveness of phage ZPAH7 reduced *A. hydrophila* by 1.5 log_10_ CFU/cm^2^. The study by You et al.^67^, reported that when the *V. parahaemolyticus* strain FORC_023 was used to artificially contaminate raw fish slices and held at 5 °C without phage treatment, a marginal decrease of the pathogen load was detected. However, when phage VPT02 was applied to the fish samples, CFU/g of FORC_023 decreased dramatically up to 1.5 log. Spraying a solution of bacteriocin AS-48 on fillets of smoked salmon reduced *Listeria* 2, 3.4, 4.5, 4.25, and 4.25 log CFU/cm^2^ in respect to the untreated control at 1, 5, 10, 15, and 30 days^68^. Findings by Soni et al.^69^ showed that when P100 phage was treated to reduce *L*. *monocytogenes* on fresh catfish fillets for 10 days at 4 °C the overall reduction of *L*. *monocytogenes* on average was 1.5 log_10_, but the authors reported a slight increase in the bacterial count after 10 days. For only one of the bacterial isolates (MDR K3) used in the fish contamination trial, a decrease in CFU level in the bacteria-only sample was observed (Supplemental Fig. 3C). This result was unexpected. This experiment was conducted in triplicate and the same trend was observed each time. It is possible that factors other than phage predation are influencing bacterial growth in this culture and thus requires further investigation.

The results reported here for chicken meat samples contaminated with MDR K3 and treated with phage AhFM11 reached up to a 2-log reduction in CFU/g, whereas the results for ATCC 35654 were reduced below the detection limit at the 96 h timepoint. A study by Thung et al.^70^ evaluated the efficacy of phage CJ01 to decontaminate mutton and chicken meat contaminated with *Campylobacter jejuni* and achieved reductions of 1.7 and 1.68 log after 48 h. In the present study, AhFM11 was found to be more effective on chicken in comparison to fish meat when contaminated with either MDR K3 or ATCC 35654. Phage concentrations had decreased in titer values at 24 h but remained almost constant up to 96 h in both fish and chicken meat samples.

At optimum temperature, the bacterial hosts usually grow faster, which promotes the replication of phage particles. Whereas at a lower temperature, the speed of phage replication is considerably decreased or halted due to the lower growth rate of their hosts^71^. The efficacy of phage particles on food substrate can be affected by the type of food matrix (i.e., liquid or solid), phage immobilization due to reduction in surface water content, and the ability of certain food-associated factors that lead to the structural degradation of phage particles^72^.

AhFM11 contains no known virulence or AMR genes and demonstrated remarkable protective efficacy in in vivo challenge models, whereby no less than 93% protection was found using three different administrative routes. It is also notable that in all food decontamination studies described above that only AhFM11 achieved reductions in bacterial counts below detectable levels. Therefore, AhFM11 warrants consideration for regulatory approval and for future field use.

## Conclusion

This is the first report on the discovery of phages possessing lytic activity against hypervirulent *Aeromonas hydrophila* (HypAh-12 and HypAh-20). Of these phages, AhFM11 was sequenced and found to have no known virulence or AMR genes. The results from in vivo studies demonstrated no less than 93% protection against vAh strains whereas the in vitro studies demonstrated that AhFM11 is highly effective as a food decontaminant. These results demonstrate that AhFM11 is a non-antibiotic alternative that can be safely used to treat *A. hydrophila* infections as well as a food decontaminant.

## Methods

### Environmental isolation, host range, one step growth curve and adsorption of phage

For the isolation of phages, *A. hydrophila* (HypAh-12 and HypAh-20) obtained from the Department of Aquatic Animal Health Management, College of Fisheries, Karnataka Veterinary, Animal and Fisheries Sciences University, Matsyanagar, Mangaluru-575002, Karnataka, India^8^ were grown overnight in TSB broth. Water samples were collected from several rivers in Karnataka, India, then centrifuged for 10 min at 10,000 *x g*. The supernatant was then filtered through a 0.45 µm membrane filter (PALL Life Sciences, Acrodisc® Syringe filter, New York). The filtrates were then put through a series of spot tests to find the lytic bacteriophages by adding 10 µL onto bacterial lawns of HypAh-12 and HypAh-20 isolates. The conventional double-layer (soft) agar overlay method was then used to confirm the presence of the lytic phages^73^. To get pure phage plaques, single plaques were picked repeatedly and eluted in SM buffer (50 mM Tris–HCl; 0.1 mM NaCl, 0.1% gelatin, 8 mM MgSO_4_, pH 7.5). Phages specific to hypervirulent *A. hydrophila* HypAh-12 and HypAh-20 named AhFM10 and AhFM11 were isolated. Using the soft agar overlay method, the titer values (PFU/mL) of the isolated phages were calculated. The isolated phages were stored at 4 °C, -20 °C, and -80 °C in aliquots of SM buffer supplemented with and without 30% glycerol for the long-term phage stability studies.

Bacteriophages AhFM10 and AhFM11 were evaluated for their host range against a diverse panel of 197 bacterial isolates. A total of 170 *Aeromonas* spp. (37 *Aeromonas hydrophila* obtained from the previously published paper from Dubey et al.^74^ and the rest were isolated in the present study) and 27 non-*Aeromonas* strains obtained from the Department of Aquatic Animal Health Management (AAHM) collection at the College of Fisheries, Mangaluru, Karnataka, India were utilized. The *Aeromonas* strains comprised *A. hydrophila* (105 isolates, including samples from various Indian states and two reference strains (ATCC 35654 and ATCC 7966)), and other *Aeromonas* species as listed in Supplemental Table 1. The 27 non-*Aeromonas* strains included various species of *Bacillus, Chromobacterium, Citrobacter, Enterobacter, Edwardsiella, Enterococcus, Klebsiella, Escherichia, Lactococcus, Salmonella, Streptococcus, Staphylococcus,* and *Vibrio*. Following the spot test method of Adams^73^, phages were assessed for host specificity based on the clarity of inhibition zones after 12 h of incubation at 28 °C. Clear zones indicated susceptibility (+), while no zones indicated resistance (−).

Based on the results obtained from host range, bacteriophage AhFM11 was selected for further characterization. The latent period and burst size of phage AhFM11 were determined using a one-step growth curve experiment. AhFM11 was separately added to *A. hydrophila* HypAh20 culture (OD_600_ = 0.8) at a multiplicity of infection (MOI) of 0.001 in 1.0 mL of sterile trypticase soy broth (TSB). After incubation at 4 °C for 30 min, the mixtures were transferred to 50 mL of sterile TSB. Samples were collected every 10 min for 120 min and immediately centrifuged (10,000 *g* for 2 min). As per the standard protocol, phage titers in the supernatants were determined at each time point using the soft agar overlay method. The burst size in one-step growth experiments was calculated by dividing the average phage titer during the plateau phase by the estimated number of initially infected cells. The latency period was estimated as the time between phage adsorption and the start of the exponential growth phase in the free phage titer curve. The phage AhFM11 adsorption efficacy was determined at MOI of 1, with the *A. hydrophila* HypAH-20 culture (OD_600_ = 0.6). A volume of 3 mL of bacteria culture was treated with 300 µL of AhFM11 at room temperature (RT) separately. Samples of 100 µL were removed from the mixture and immediately diluted in 900 µL of SM buffer at regular time intervals (0, 2, 4, 6, 8, 10, 15, 20, and 30 min). Samples were vortexed and then centrifuged for 2 min at 10,000 ×*g* at room temperature. Then the free phage titers in the supernatant were examined by the soft agar overlay method^12^.

### Transmission electron microscopy (TEM) and phage stability assays of AhFM11

Phage AhFM11 was isolated from subsequent passages using the soft agar overlay method^73^. The lysate containing the phage particles was then filtered through a 0.45 µm membrane filter (PALL Life Sciences, Acrodisc® Syringe filter, New York) to remove bacterial cells and retain only the phage particles. The purified phage (AhFM11) was ultracentrifuged at 24,500 × *g* for 3 h at 4 °C (Hitachi, Himac CS150GXII, Japan) to obtain high titer purified phages (10^12^⁻^14^ PFU/mL). The high titer purified phages were then washed twice with 0.1 M ammonium acetate. About 10 µL of washed high titer purified phage were placed on a carbon-coated 400-mesh TEM copper grid (NisshinME, Tokyo, Japan) and then negatively stained with 2% uranyl acetate for 5 min. Imaging was performed using a Technai G2 T12 BioTwin TEM (Hillsboro, USA) at 160,000 × magnification and 120 kV at IISc, Bengaluru, Karnataka, India.

In order to check the efficacy of the phage AHFM11 at different environmental conditions, phage was stored at different temperature and salinity for 60 days; pH and chemicals for 24 h were used^12^. Temperature stability was assessed by storing 1 mL aliquots (10^12^ PFU/mL) at − 80 °C (with (WG) and without glycerol (WoG)), -20 °C (WG and WoG), 0 °C, 4 °C, 28 °C, and 37 °C for 60 days. Phage titers were determined every 15 days using the soft agar overlay method by using aliquots of phage AhFM11 (100 µL). Salinity stability was examined by storing 1 mL aliquots in varying salinity solutions (0.1%, 0.5%, 1.0%, 2.0%, 3.5%) under refrigerated conditions for 60 days. Phage titers were similarly determined every 15 days by soft agar overlay method. Stability across a pH range (2–12) and against various chemicals (acetone, chloroform, diethyl ether, ethanol, phenol, propanol) was also investigated. For pH, 100 µL phages were incubated in SM buffer (900 µL) at each pre-defined pH for 10 min, 12 h, and 24 h. Similarly, 100 µL phages were incubated with each solvent (900 µL) for the same time intervals. Surviving phages were enumerated at 10 min, 12 h and 24 h time point using the soft agar overlay method.

### Whole genome sequencing of AhFM11 and phylogenetic analysis

About 50 mL of the purified phages (10^11–12^ PFU/mL) were concentrated by centrifugation (10,000 x *g* for 10 min) and again filtrated (0.45 µm). The filtrate was treated with DNase I (100 µg/mL) and RNase A (20 µg/mL) (Himedia, India) at 37 °C for 1 h to remove bacterial nucleic acids. Phages were then precipitated overnight at 4 °C with 0.4 volumes of a precipitation solution [3.3 M NaCl and 30% Polyethylene Glycol (PEG 6000)]. The overnight kept phages were centrifuged at 15,000 x *g* for 30 min at 4 °C. The pellet was resuspended in 500 µL phage lysis buffer (100 mM NaCl, 50 mM Tris–HCl, 0.4% Sodium dodecyl sulfate (SDS), 100 mM EDTA, pH 8.0) and treated with proteinase K (100 µg/mL) at 60 °C for 1 h. This mixture was treated with an equal volume of phenol:chloroform:isoamyl alcohol (25:24:1 v/v) twice and absolute ethanol was used to precipitate the phage nucleic acid. The nucleic acid pellet was again washed with 70% ethanol. Then again centrifuged at 10,000 × *g* for 10 min and the supernatant was discarded. The pellet was finally dissolved in TE buffer (1 mM EDTA, 10 mM Tris–HCl, pH 8.0). Concentration and purity of DNA was measured in a NanoDrop spectrophotometer (Thermo Fisher Scientific, USA) and using gel electrophoresis.

Isolated phage genomic DNA of AhFM11 was sequenced at Clevergene Biocorporation Private Limited located in Bengaluru, Karnataka, India. Sequencing was performed on an Illumina HiSeq using 2 × 150 bp paired-end configuration. To assess the data quality of the obtained sequence, Fast QC version 0.11.2 and Multi QC version v1.11 was used to check the quality of sequenced data. Sequencing adapter contamination and other low-quality reads were removed by using Trim Galore version 0.6.7. De novo assembly was performed using SPAdes version 3.12.0. The complete assembled sequence was aligned in NCBI using BLASTn. Prokka version 1.14.6 was used to annotate the genome and identify features of interest. A circular map of the AhFM11 whole genome was constructed using the Proksee online tool^33^. The presence of virulence and antibiotic resistance genes in the genome was checked by Virulence Factors of Pathogenic Bacteria Database (VFDB) and the Resistance Gene Identifier (RGI) of the Comprehensive Antibiotic Resistance Database (CARD). The genome was visualized using the CG view server. To determine the lytic or lysogenic life cycle of phage, Phage Classification Tool Set (PHACTS) software was used. Comparative genomics were conducted using the Easyfig tool^75^. Phylogenetic analysis of the major capsid proteins was performed using software MEGA XI version 11.0^34^. Whole-genome sequences of phages were aligned using MAFFT v7.3^76^; then, a phylogenetic tree was constructed using the iTOL (https://itol.embl.de/)^77^.

### In vivo experiments with phage AhFM11 against *Aeromonas* infection

All experiments were conducted in the Department of Aquatic Animal Health Management wet lab, College of Fisheries, Mangaluru, Karnataka, India. The fish used in these phage therapy experiments were rohu carp (*Labeo rohita*) with a 7.7 to 9.8 g body mass and 6.7 cm average length, obtained from BRP, Shivamogga, Karnataka, India. The fish had not been vaccinated or exposed to disease and were healthy without any symptoms of infection. All fish were fed a basal diet during four to five weeks of acclimatization.

To establish the LD_50_ for vAh (HypAh-20) used in challenge models, rohu carp (*Labeo rohita*) were divided into challenged and unchallenged groups (n = 30) and received intraperitoneal injections of either PBS + SM buffer in the unchallenged group or vAh (HypAh-20) in challenged groups. Fish were observed for 15 days post infection (dpi). The LD_50_ was determined to be 2.42 × 10^6^ CFU/mL with the final cumulative survival of 100% in the unchallenged and 43.33% in the challenged group.

During the experiment, the temperature, the level of dissolved oxygen and pH were monitored and controlled. The fish were fed a commercial fodder using automatic band feeders in an amount suitable for body weight and temperature. Physicochemical conditions were maintained at temperature (25–28 °C), dissolved oxygen (5–8 mg/L) and pH (6.5–7.5). A total of 150 fish were categorized into five groups and challenged with vAh (HypAh-20). The five groups consist of the Injection Group, Immersion Group, Oral Feeding Group, Negative Control (without phage but challenged), and Positive Control (without phage and without challenge) (Supplemental Fig. 5).

For the in vivo phage efficacy tests, bacteriophage AhFM11 with a titer value of 10^10^ PFU/mL was used as a stock solution and respective dilutions were made for the treatment groups. For the Injection and Immersion experimental groups, fish received an intraperitoneal injection containing an LD_50_ of vAh (HypAh-20) 2-h prior to phage administration. The fish in the Injection group received an intraperitoneally injection containing 1.55 × 10^6^ PFU/fish 2-h post infection. Fish within the Immersion group were kept in water for 15 min with phage at a concentration of 1.55 × 10^7^ PFU/mL 2 h post infection. For the Oral Feeding group, phage AhFM11 at a concentration of 1.55 × 10^7^ PFU/g of feed pellets was thoroughly mixed in sterile 50 mL tubes by uniformly spraying on the surface and then evaporated in a sterile hot air oven cabinet for 30 min. The fish were continuously fed phage top-coated feed beginning 60-days before challenge and throughout the experiment. The control groups were treated in the same manner but with PBS in place of phage. The fish were monitored every day, and mortalities were removed immediately. Mortality was recorded daily for 15 days, and cumulative percent survival rate was calculated. The cause of mortality was confirmed by re-isolating the bacteria from the kidney of dead fish using selective *Aeromonas* isolation media base (AIMB).

### Food decontamination efficacy of phage AhFM11 on *Aeromonas* hydrophila

To test the efficacy of AhFM11 as a food decontamination tool, chicken and fish meats were artificially contaminated with 10^6^ colony forming unit (CFU)/mL of multidrug resistant (MDR) *A. hydrophila* (K3) or ATCC 35654 at room temperature. AhFM11 was added at an MOI = 1000 (10^9^ PFU/mL) 30 min after the bacteria and the samples were stored at 4 °C for 4 days. The bacteria were aseptically recovered from both phage treated and untreated meat samples. Multidrug resistance (MDR) K3 *A. hydrophila* isolate and American Type Cell Culture 35654 (ATCC 35654) were used in this study. These two bacteria were available in the stock culture of the Department of Aquatic Animal Health Management (AAHM), College of Fisheries, Mangaluru, Karnataka, India. To prepare an *A. hydrophila* culture, a single colony was used to inoculate Luria Bertani broth (LB broth) and incubated at 28°C overnight.

The efficiency of phage AhFM11 was tested on fish meat and chicken meat which was artificially contaminated with MDR K3 and ATCC 35654 *A*. *hydrophila* and stored at a refrigerated storage temperature of 4 °C for 96 h incubation. Fresh raw fish and chicken fillets were procured from local markets. The samples were then placed in a cooler box containing ice packs and transported to the laboratory, Department of Aquatic Animal Health Management (AAHM) College of Fisheries, Mangaluru, Karnataka, India, and the meat was processed within an hour of collection. The samples were aseptically cut into 1 cm^3^ (0.5 ± 0.02 g) using sterile scissors and forceps and then transferred into sterile trays containing an average meat mass of 10 ± 0.2 g. The trays were held under UV lamps for 30 min inside a laminar flow hood to kill any contaminating bacteria.

The chicken and fish meat samples were divided into treatment groups as mentioned in Table 2. Groups within these studies containing bacteria were artificially contaminated with either MDR K3 or ATCC 35654 *A*. *hydrophila* by directly pipetting 2 mL (10^6^ CFU/mL) of bacterial suspension onto the surface of fish and chicken meat samples in separate sterile 50 mL tubes. Samples were held at room temperature for 30 min inside a laminar flow hood to allow for bacterial attachment to the sample surface. Next, 2 mL of phage AhFM11 at a concentration of 10^9^ PFU/mL (MOI = 1000) was pipetted directly on the meat surface and kept at room temperature inside the laminar flow hood for 30 min. For the Control groups, 2 mL of SM buffer was added in place of the phage suspension. The sample tubes were wrapped with parafilm to avoid contamination and refrigerated at 4 °C for 4 days. At timepoints 0, 24, 48, 72, and 96 h post-inoculation, 2.5 ± 0.2 g from each sample group were transferred to stomacher bags and homogenized. A 10 mL samples of homogenate were transferred into 15 mL sterile tubes and centrifuged at 12,000 ×*g* at 4 °C for 10 min to pellet the bacteria. Pellets were resuspended in TSB broth, pH 7.5. Tenfold serial dilutions were prepared in TSB broth, and aliquots were spread onto selective AIMB supplemented with ampicillin (HiMedia, Mumbai) for quantification of bacteria as CFU/g of meat samples.

### AhFM11 replication and longevity on meat

Fish and chicken meat samples were prepared as described above and divided into groups as mentioned in Table 3. In this experiment, 2 mL of AhFM11 was added at a concentration of 10^9^ PFU/mL to their respective groups. For negative controls, 2 mL of SM buffer was added instead of the phage suspension and 2 mL of TSB broth was added in place of bacteria. Meat samples were processed in stomacher bags and centrifuged as described above. The supernatant containing phage was collected and serially diluted in SM buffer to quantify the phage as PFU/g of meat using the soft agar overlay method described above.

**Table 3:**
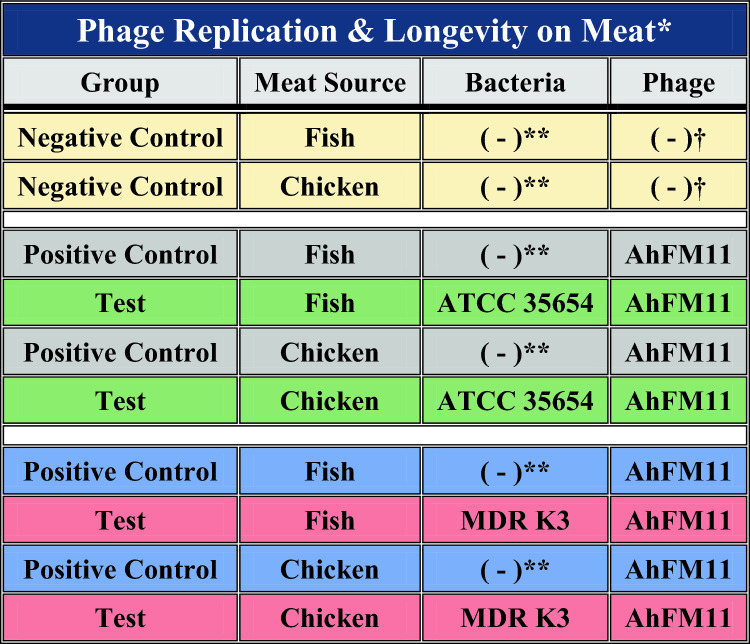
Food decontamination efficacy of phage AhFM11 with different treatment groups in fish and chicken meat to test the phage replication and longevity.

*Colors are coordinated to match Supplemental Fig. 4.

**TSB broth added in place of Bacteria.

^†^SM buffer used in place of bacteriophages.

### Statistical analysis

One-way analysis of variance (ANOVA) was used to analyze the data along with Tukey's HSD (honestly significant difference) test using IBM SPSS Statistics 20 (SPSS, Chicago, IL, USA). The difference between the treatments was determined by an independent t-test. Statistically significant differences were considered at *p* < 0.05 and the data were represented as mean ± standard error of the mean using GraphPad Prism software.

### Ethical statement

This study is reported in accordance with ARRIVE guidelines (https://arriveguidelines.org). The authors have adhered to all relevant international, national and/or institutional guidelines for animal care and animal utilization approved by the Institutional Animal Ethics Committee, College of Fisheries, Mangaluru, India. The experiments conducted on rohu (*Labeo rohita*) is a commercial fish which is not an endangered species in India.

### Supplementary Information

Supplementary Legends.

Supplementary Figure 1.

Supplementary Figure 2.

Supplementary Figure 3.

Supplementary Figure 4.

Supplementary Figure 5.

Supplementary Table 1.

Supplementary Table 2.

## Data Availability

The datasets generated and/or analyzed during the current study are available in the NCBI repository, accession number ON042478.1.
