# Supplementary material for: Novel lytic bacteriophage AhFM11 as an effective therapy against hypervirulent Aeromonas hydrophila
Source: Sci Rep. 2024 Jul 23;14:16882. doi: 10.1038/s41598-024-67768-2 (PMC11266544; doi:10.1038/s41598-024-67768-2)
Supplement: Supplementary file 1 — Supplementary Legends. [file 41598_2024_67768_MOESM1_ESM.docx]

**Supplementary Data**

**Supplemental Figure 1.** Map depicting the isolation sites of phages AhFM10 and AhFM11. These phages were isolated from geographically distinct locations and subsequently evaluated for their efficacy against hypervirulent *Aeromonas hydrophila* HypAh-20. Map was created using Tableau version 2019.3.0 (www.tableau.com). The data used to create the map was sourced from Latitude and Longitude (AhFM10 -14.646 and 74.321; AhFM11 -14.448 and 74.446), located in the Karnataka State, India.

**Supplemental Figure 2.** Stability tests of AhFM11 phages under various conditions:

(A) Different temperatures (-80°C, -20°C, 0°C, 4°C, 28°C, and 37°C).

(B) Different salinity levels (0.1%, 0.5%, 1.0%, 2.0%, and 3.5%), with glycerol (WG) and without glycerol (WOG) for a period of 60 days, measured as log PFU/mL of viable phages.

(C) Different pH levels (2, 4, 5, 6, 7, 8, 10, and 12).

(D) Various organic solvents (Phenol, chloroform, propanol, SM buffer, PBS, ethanol, diethylether, acetone) for a period of 24 hours, measured as log PFU/mL of viable phages.

Experiments were conducted in triplicates, with error bars representing the mean ± SD. Statistical significance is indicated by asterisks (*) for differences with p < 0.05. Statistical analyses were performed using one-way analysis of variance (ANOVA) followed by Tukey's HSD (honestly significant difference) test.

**Supplemental Figure 3** Ten-gram samples of fish or chicken meat were contaminated with 1012 CFUs of either *A. hydrophila* MDR K3 or ATCC 35654 at room temperature. Phage AhFM11 was added at an MOI = 1,000 thirty minutes after the bacteria and the samples were stored at 4°C for 4 days. At each 24-hour timepoint, a sample was removed and plated for CFU counts.

(A) Fish meat contaminated with ATCC 35654

(B) Chicken meat contaminated with ATCC 35654

(C) Fish meat contaminated with MDR K3

(D) Chicken meat with MDR K3. Negative growth controls yielded no colonies and are not depicted above. Error bars depict the standard error of the mean (n=3).

**Supplemental Figure 4.** AhFM11 was applied to 10g samples of fish or chicken meat with or without bacteria. At each 24-hour timepoint, a sample was removed and used in soft agar overlay plaque assays.

**(A)** Fish meat contaminated with ATCC 35654

**(B)** Chicken meat contaminated with ATCC 35654

**(C)** Fish meat contaminated with MDR K3

**(D)** Chicken meat with MDR K3. Negative growth controls yielded no colonies and are not depicted above. Error bars depict the standard error of the mean (n=3).

**Supplemental Figure 5.** Experimental design followed for the administration of the phage AhFM11 in rohu (*Labeo rohita*) by injection, immersion, and phage impregnated feed by spraying the phage on surface of pelleted feed.

**Supplemental Table 1.** The results of the spot test on the collected bacterial isolates to determine the host range of the isolated phages AhFM10 and AhFM11.

**Supplemental Table 2.** The list of genes, location, coding sequences (CDS), gene name and length (bp) in phage genome AhFM11.
