# Supplementary figures and images for: Novel lytic bacteriophage AhFM11 as an effective therapy against hypervirulent Aeromonas hydrophila
Source: Sci Rep. 2024 Jul 23;14:16882. doi: 10.1038/s41598-024-67768-2 (PMC11266544; doi:10.1038/s41598-024-67768-2)

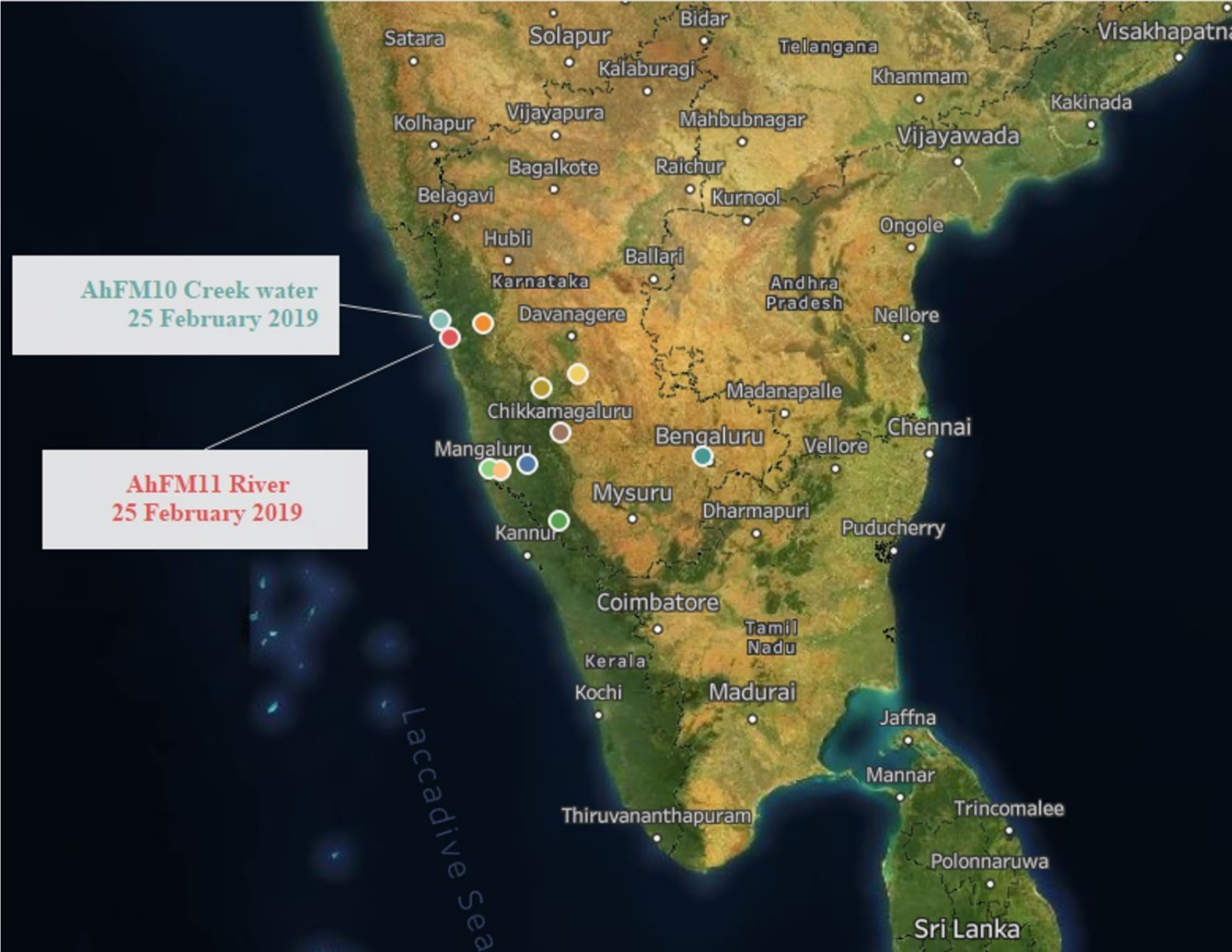

Supplement: Supplementary file 2 — Supplementary Figure 1. [file 41598_2024_67768_MOESM2_ESM.pdf]

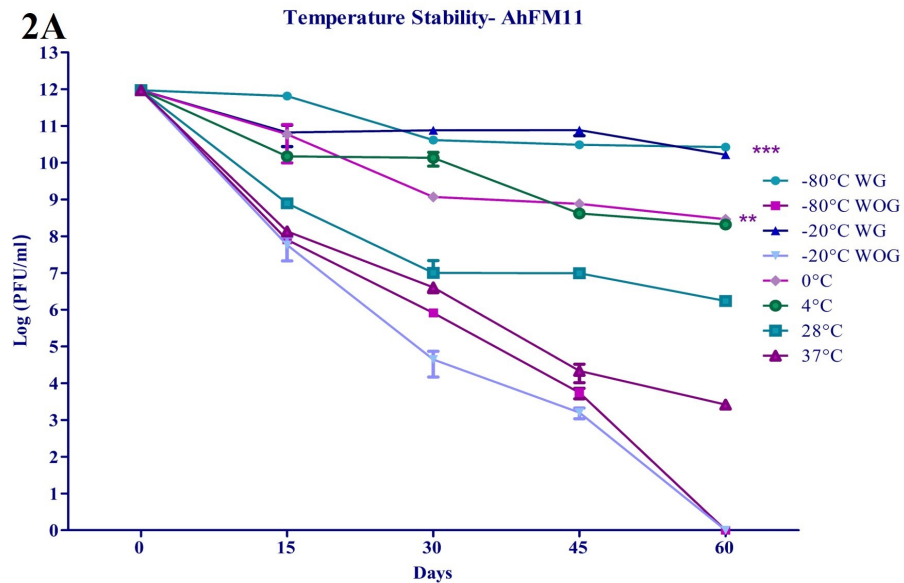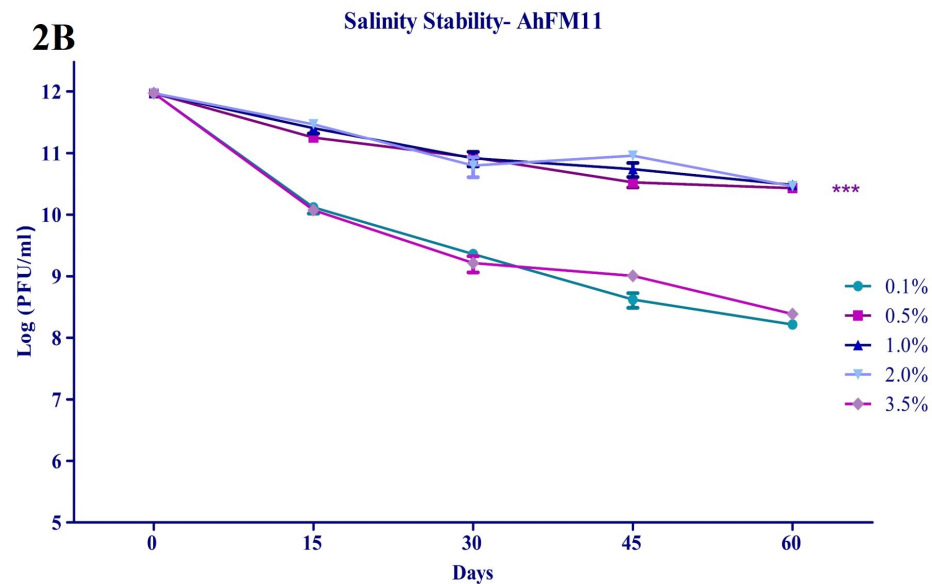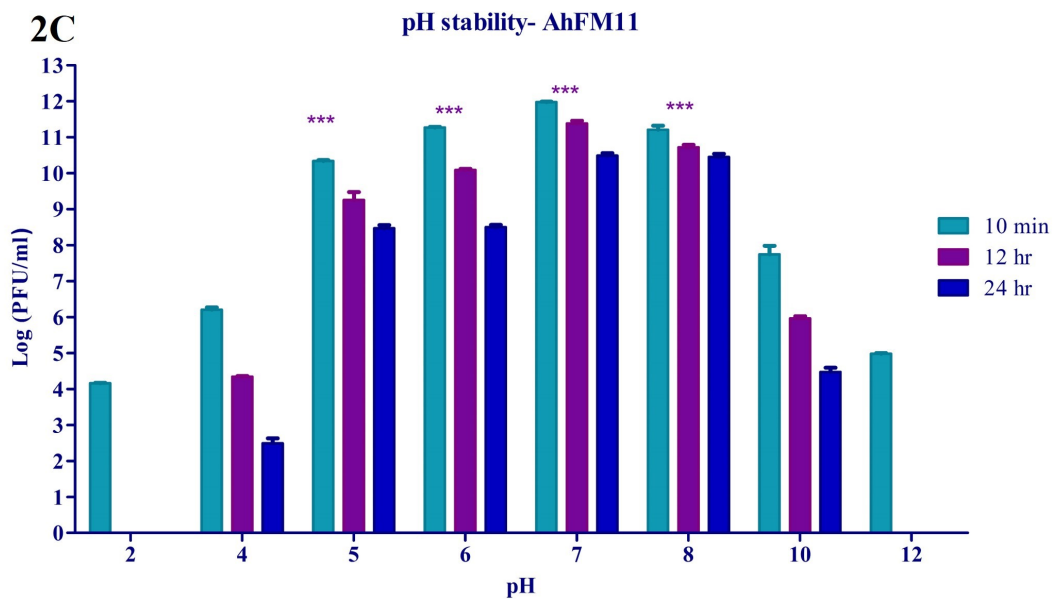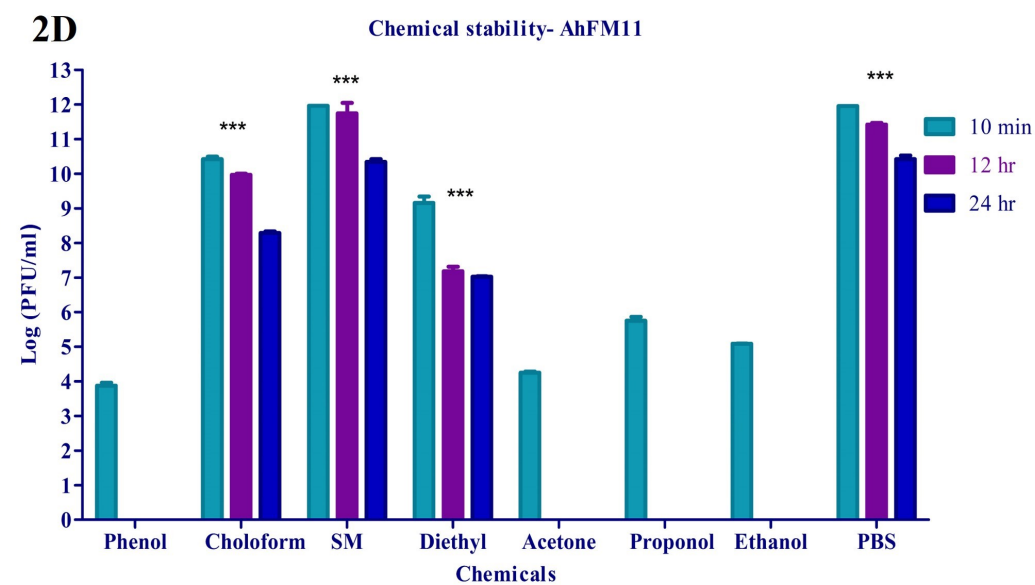

Supplement: Supplementary file 3 — Supplementary Figure 2. [file 41598_2024_67768_MOESM3_ESM.pdf]

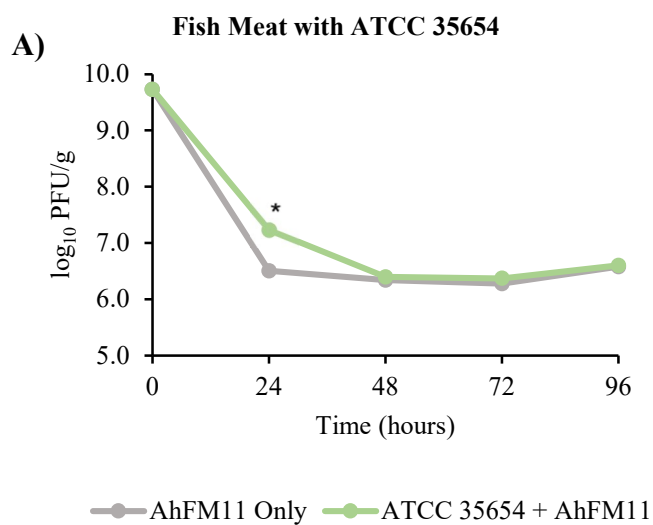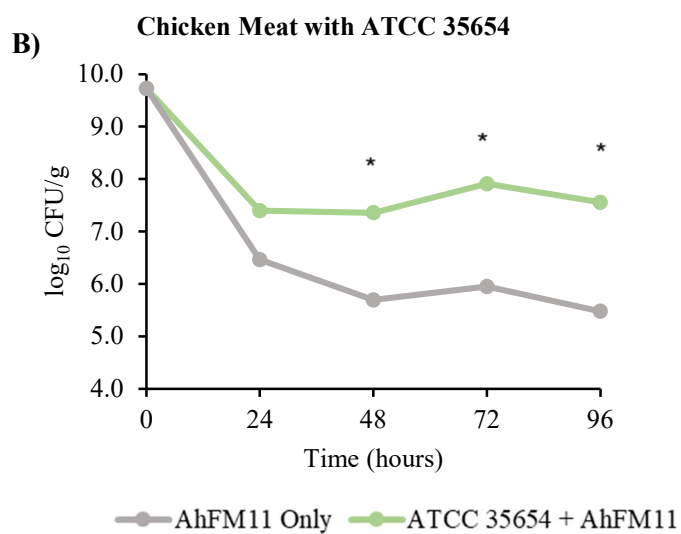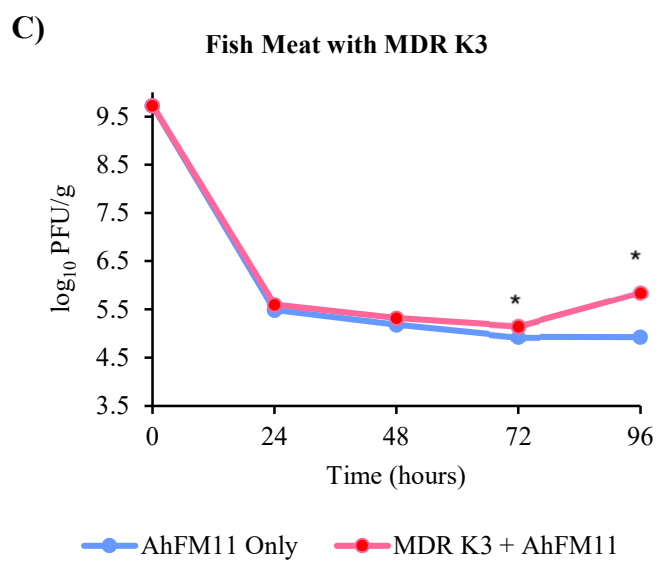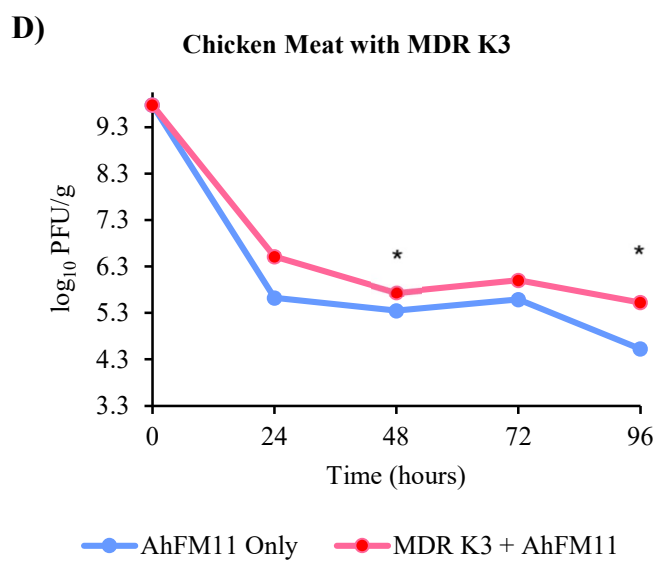

Supplement: Supplementary file 5 — Supplementary Figure 4. [file 41598_2024_67768_MOESM5_ESM.pdf]
