# Supplementary material for: Novel lytic bacteriophage AhFM11 as an effective therapy against hypervirulent Aeromonas hydrophila
Source: Sci Rep. 2024 Jul 23;14:16882. doi: 10.1038/s41598-024-67768-2 (PMC11266544; doi:10.1038/s41598-024-67768-2)
Supplement: Supplementary file 4 — Supplementary Figure 3. [file 41598_2024_67768_MOESM4_ESM.pdf]

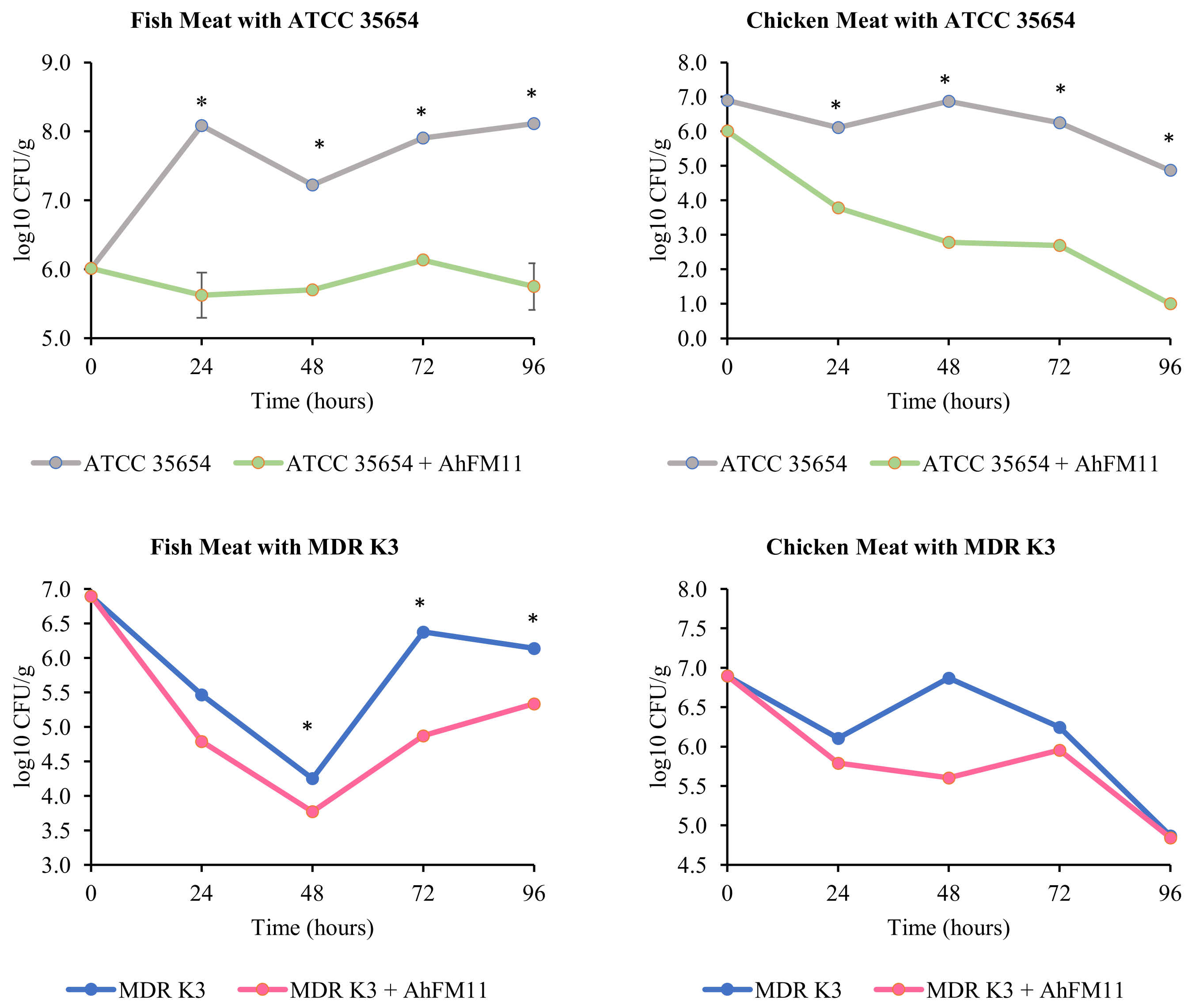
Fish Meat with ATCC 35654
log10 CFU/g
9.0
8.0
7.0
6.0
5.0
0
24
48
72
96
Time (hours)
ATCC 35654
ATCC 35654 + AhFM11
Chicken Meat with ATCC 35654
log10 CFU/g
8.0
7.0
6.0
5.0
4.0
3.0
2.0
1.0
0.0
0
24
48
72
96
Time (hours)
ATCC 35654
ATCC 35654 + AhFM11
Fish Meat with MDR K3
log10 CFU/g
7.0
6.5
6.0
5.5
5.0
4.5
4.0
3.5
3.0
0
24
48
72
96
Time (hours)
MDR K3
MDR K3 + AhFM11
Chicken Meat with MDR K3
log10 CFU/g
8.0
7.5
7.0
6.5
6.0
5.5
5.0
4.5
0
24
48
72
96
Time (hours)
MDR K3
MDR K3 + AhFM11
