# Supplementary material for: Novel lytic bacteriophage AhFM11 as an effective therapy against hypervirulent Aeromonas hydrophila
Source: Sci Rep. 2024 Jul 23;14:16882. doi: 10.1038/s41598-024-67768-2 (PMC11266544; doi:10.1038/s41598-024-67768-2)
Supplement: Supplementary file 6 — Supplementary Figure 5. [file 41598_2024_67768_MOESM6_ESM.pdf]

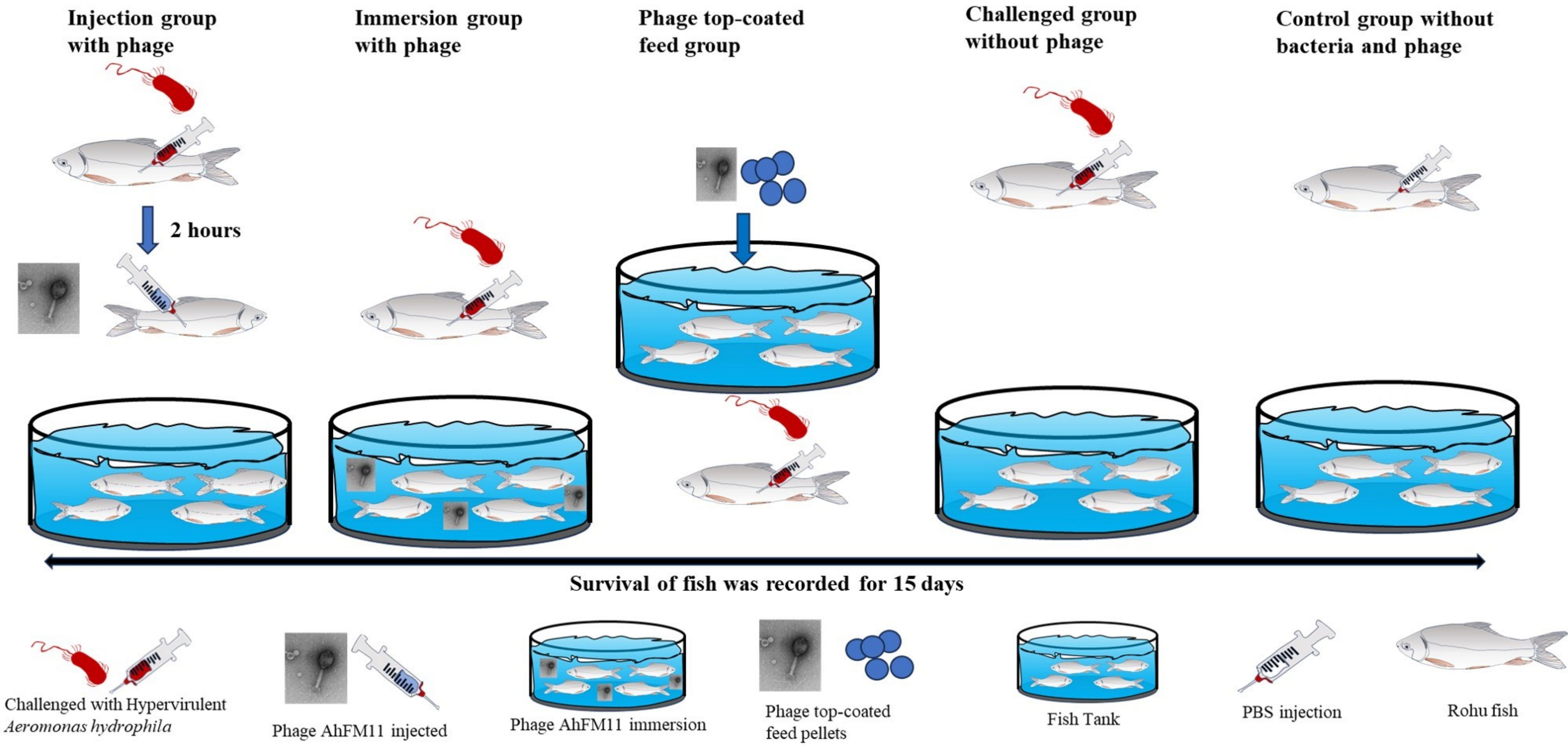

Injection group with phage
Immersion group with phage
Phage top-coated feed group
Challenged group without phage
Control group without bacteria and phage
2 hours
Survival of fish was recorded for 15 days
Challenged with Hypervirulent *Aeromonas hydrophila*
Phage AhFM11 injected
Phage AhFM11 immersion
Phage top-coated feed pellets
Fish Tank
PBS injection
Rohu fish
